# Beyond single time point self‐report: EMA as a tool for understanding mood and cognition in older adults without dementia

**DOI:** 10.1002/alz70857_104567

**Published:** 2025-12-25

**Authors:** Sophia L. Holmqvist, Riya Chaturvedi, Marina Kaplan, Tania Giovanetti

**Affiliations:** ^1^ Temple University, Philadelphia, PA, USA

## Abstract

**Background:**

Depressive symptoms are strongly linked to self‐reported cognitive decline in older adults. Gold‐standard assessments of depression and functional abilities are typically measured at one time point, but EMA can efficiently capture overall and fluctuations in daily mood and subjective cognition. Understanding daily fluctuations and interactions among mood and cognition may reveal patterns missed by gold‐standard assessment. This study sought to explore the relations among cognition, daily EMA mood and subjective cognition, and conventional self‐report measures of depression and function.

**Method:**

Fifty‐five community dwelling older adults with healthy cognition (*n* = 45; 30.9% Black/African American) or mild cognitive impairment (MCI; n = 10) completed a daily EMA smartphone‐based survey for 30 days that assessed self‐reported mood (happy, neutral, sad) and subjective cognition (very sharp, neutral, not sharp). EMA metrics were converted to percentages of total days of EMA data. Baseline measures included global and domain cognitive composites computed from demographically‐ adjusted cognitive tests, self‐reported depression [Geriatric Depression Scale‐15 (GDS)], cognitive decline [Measurement of Everyday Cognition (Ecog)], and function [Functional Activities Questionnaire (FAQ)]. Spearman correlations were run to examine relations among EMA mood, subjective cognition, conventional tests of cognition, and self‐reported baseline depression and cognitive decline.

**Result:**

Lower percent of “happy” days and greater percent of “neutral” mood days were related to higher GDS (ρ = ‐.385, *p* = .004; ρ = .416, *p* = .002). Neither EMA mood ratings nor the GDS were related to the cognitive composite. A greater percent of “not sharp” days was related to greater self‐reported cognitive decline and functional difficulties (FAQ and Ecog) (ρ = .357, *p* = .009; ρ = .309, *p* = .002). Greater percent of “not sharp” days (and ECog) were significantly associated with a lower cognitive composite (ρ=‐.279, *p* = .039; ρ=‐.319, *p* = .018) and higher GDS score (ρ = .408, *p* = .002; ρ = .384, *p* = .004)

**Conclusion:**

Daily EMA mood and subjective cognition measures were significantly related to conventional clinical measures of mood, cognitive decline, and everyday function in expected directions. Subjective cognition (EMA and conventional measures) but not mood (EMA and GDS) was related to objective cognition. Results support the validity of conventional self‐report measures and suggest EMA is a valid method for assessing older adults’ mood and cognition “in the wild”.

